# Development of International Practice Recommendations for Persons With Hearing, Vision, and Cognitive Impairment

**DOI:** 10.1093/geroni/igab046.328

**Published:** 2021-12-17

**Authors:** Barbara Weinstein, Jenna Littlejohn, Iracema Leroi

**Affiliations:** 1 City University of New York, New York, New York, United States; 2 University of Manchester, Manchester, England, United Kingdom; 3 Global Brain Health Institute, Dublin, Dublin, Ireland

## Abstract

Many older adults being evaluated for dementia have unrecognized hearing and/or vision problems which can confound results of neuropsychological assessment(s) and can impact care recommendations. International care standards for detection, assessment, and management of people living with dementia (PwD) are rarely addressed yet are critical. We propose a set of recommendations crafted to foster the highest quality health care to enable PwD to live well with these combined impairments. The focus is detection, diagnosis, treatment, and support of PwD who have age-related hearing and/or vision impairments. The guiding principles underlying the recommendations was a focus on promotion of a person-centered approach, but to be pragmatic in considering all contextual levels including professional care pathways and socio-economic/policy factors internationally. The recommendations are inclusive of all stakeholders who work together to promote equity and mutual respect across the domains. The guidelines are designed to be pragmatic, implementable, resource sparing, and sustainable.

